# Gingival Bleeding in a Heavy Drinker

**DOI:** 10.7759/cureus.71133

**Published:** 2024-10-09

**Authors:** Masaya Iwamuro, Tomohiro Kamio, Motoyuki Otsuka

**Affiliations:** 1 Gastroenterology and Hepatology, Okayama University Hospital, Okayama, JPN; 2 Gastroenterology and Hepatology, Okayama University Graduate School of Medicine, Dentistry, and Pharmaceutical Sciences, Okayama, JPN

**Keywords:** alcoholic, esophagogastroduodenoscopy, gingival bleeding, oral lesions, periodontal disease

## Abstract

This case report presents a 34-year-old Japanese man with hematemesis who was ultimately diagnosed with gingival bleeding due to periodontitis via esophagogastroduodenoscopy. Although endoscopic examination revealed no gastrointestinal lesions, persistent bleeding from the gums was observed, highlighting the need to consider non-gastrointestinal sources of bleeding in such scenarios. Heavy alcohol consumption likely contributed to poor oral health and increased the risk of oral bleeding. This case emphasizes the importance of thorough endoscopic evaluation of the oral and pharyngolaryngeal regions, particularly when common gastrointestinal sources of bleeding are excluded. A comprehensive diagnostic approach is essential to avoid overlooking less common causes of bleeding.

## Introduction

Hematemesis, or vomiting of blood, is a potentially life-threatening condition requiring immediate evaluation and intervention. It is most commonly associated with upper gastrointestinal bleeding, which can be caused by conditions such as esophageal or gastric varices, peptic ulcers, or Mallory-Weiss tears. These conditions are particularly prevalent in individuals with risk factors such as heavy alcohol consumption or liver cirrhosis, making gastrointestinal sources the initial focus during diagnostic evaluations [1-3]. The standard diagnostic approach for hematemesis typically involves esophagogastroduodenoscopy, which allows direct visualization of the esophagus, stomach, and duodenum to identify the source of bleeding. However, in some cases, no gastrointestinal lesions are found, prompting the need to consider alternative, non-gastrointestinal sources of bleeding.

Non-gastrointestinal sources, such as oral bleeding, may initially be overlooked, especially in patients presenting with symptoms suggestive of a gastrointestinal origin. One such non-gastrointestinal cause is gingival bleeding, often related to periodontitis, a severe gum infection that damages the soft tissue. The link between heavy alcohol consumption and poor oral health is well-documented [4-6]. Chronic alcohol abuse is also associated with immune system suppression and nutritional deficiencies, further exacerbating oral health problems and raising the likelihood of bleeding.

This case report presents a patient who experienced hematemesis, where endoscopic evaluation revealed no gastrointestinal lesions. Instead, the bleeding was ultimately traced to the gums, and the patient was diagnosed with gingival bleeding. This case highlights the importance of considering non-gastrointestinal sources, particularly oral lesions, when investigating hematemesis, especially in individuals with risk factors like alcohol abuse.

## Case presentation

A 34-year-old Japanese man was urgently transported to the hospital with the chief complaint of hematemesis. The patient did not experience any oral symptoms. He had a history of heavy alcohol consumption. He was previously diagnosed with transient ascites due to alcoholic liver disease at another hospital. However, he did not receive regular follow-up care at any medical institution and was not taking any medications, including antithrombotic agents. The patient was also not taking any supplements. The patient began vomiting at 10 pm the previous day and started spitting blood at 4 am, leading to emergency transport to our hospital. Upon arrival, the vital signs were as follows: heart rate of 130bpm, blood pressure of 135/80mmHg, respiratory rate of 20/min, oxygen saturation of 100%, and body temperature of 36.0°C. Physical examination revealed mild anemia and conjunctival jaundice. His abdomen was flat and soft, with no tenderness. There were no signs of ascites or engorged abdominal veins. Blood tests revealed normocytic anemia, with a red blood cell count of 4.02×10^6^/μL, a hemoglobin level of 12.7g/dL, and a hematocrit count of 37.1% (Table 1). Thrombocytopenia was also observed, with a platelet count of 129×10^3^/μL. The levels of liver and biliary enzymes were elevated: aspartate aminotransferase, 175 U/L; alanine aminotransferase, 68U/L; gamma-glutamyl transpeptidase, 1,667U/L; lactate dehydrogenase, 269U/L (normal range: 124-222U/L); and alkaline phosphatase, 173U/L.

**Table 1:** Blood test results

Blood test results (units)	Patient value	Reference range
White blood cells (/μL)	9,610	3,300–8,600
Neutrophil (%)	75.1	40–70
Lymphocyte (%)	20.5	16.5–49.5
Monocyte (%)	3.6	2–10
Red blood cells (/μL)	4.02×10^6^	4.35×10^6^–5.55×10^6^
Hemoglobin (g/dL)	12.7	13.7–16.8
Hematocrit (%)	37.1	40.7–50.1
Platelets (/μL)	129×10^3^	158×10^3^–348×10^3^
Total protein (g/dL)	7.4	6.6–8.1
Albumin (g/dL)	4.3	4.1–5.1
Creatinine (mg/dL)	0.76	0.65–1.07
Sodium (mmol/L)	137	138–145
Potassium (mmol/L)	3.3	3.6–4.8
Total bilirubin (mg/dL)	2.68	0.4–1.5
Direct bilirubin (mg/dL)	0.98	0.08–0.28
Aspartate aminotransferase (U/L)	175	13–30
Alanine aminotransferase (U/L)	68	10–42
γ-Glutamyl transpeptidase (U/L)	1667	38–113
Lactate dehydrogenase (U/L)	269	124–222
Alkaline phosphatase (U/L)	173	38–113
C-reactive protein (mg/dL)	0.02	0–0.15
D-dimer (μg/mL)	<0.5	0–0.9
Prothrombin time (%)	77	73–118
Activated partial thromboplastin time (sec)	24.5	24–34
Ammonia (μg/dL)	12	15–66

Additionally, a total bilirubin level of 2.68mg/dL indicated jaundice. Serum ammonia levels, prothrombin levels, and activated partial thromboplastin time were within normal ranges. Because the consultation occurred in the emergency department, no further tests for bleeding tendencies could be conducted.

**Figure 1:**
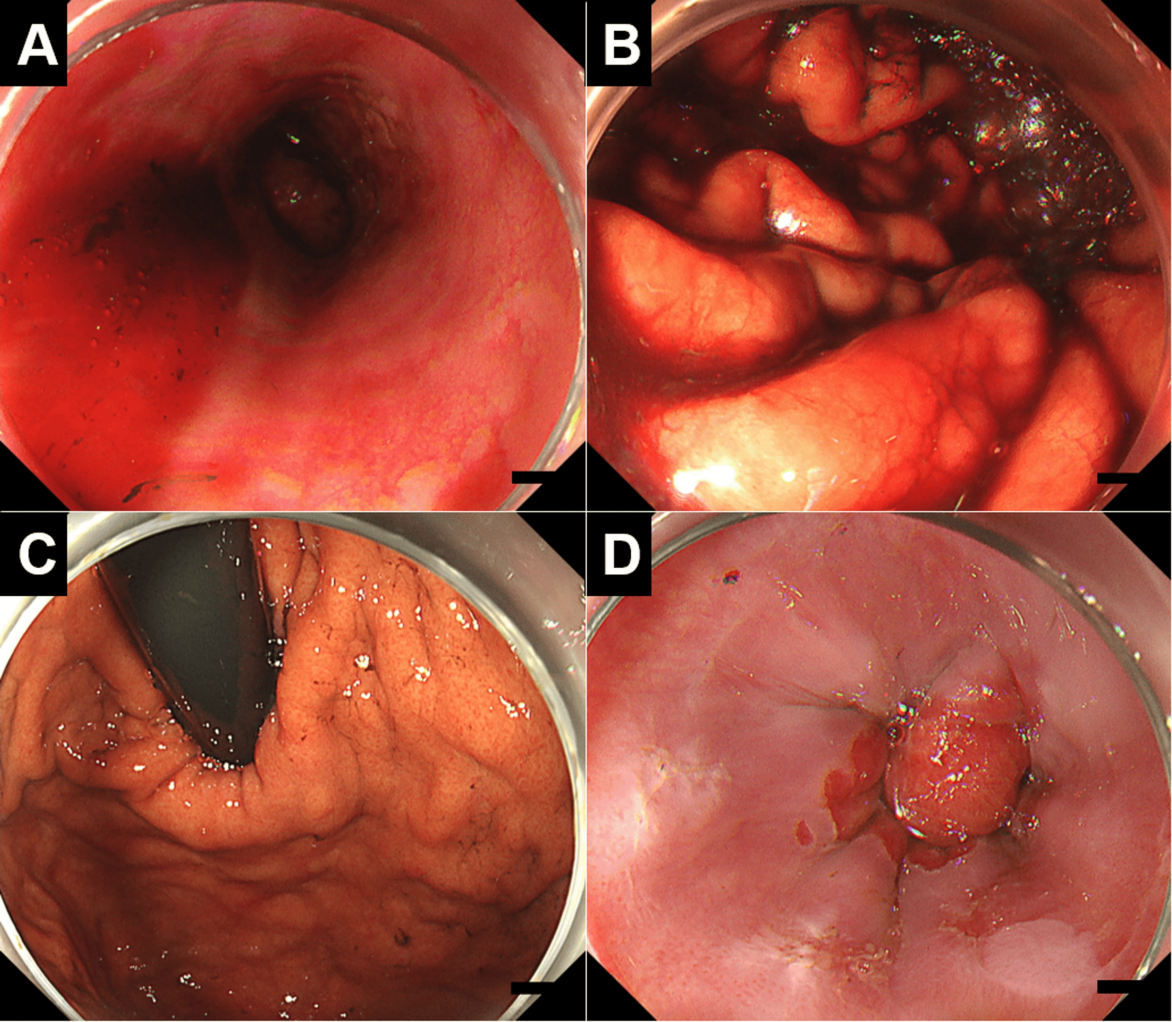
Esophagogastroduodenoscopy images of the esophagus and stomach Fresh blood pooling was observed in the esophagus (A) and stomach (B). When fresh blood is seen in the esophagus, stomach, or duodenum, bleeding from these regions is typically suspected. However, subsequent suctioning and removal of the blood did not reveal any varices, erosions, ulcers, or vascular ectasia in either the stomach (C) or esophagus (D). The duodenum was also found to be intact. In such cases, the possibility of bleeding from lesions that are difficult to visualize endoscopically, such as a small Dieulafoy ulcer that has stopped bleeding spontaneously, or bleeding from sources outside the esophagus, stomach, or duodenum, such as nasal or oral cavity bleeding, must be considered.

Esophagogastroduodenoscopy was promptly performed to identify the source of bleeding, with suspected conditions including esophageal or gastric varices, peptic ulcers, and Mallory-Weiss tears. Pooling of fresh blood was observed in the esophagus (Fig 1A) and stomach (Fig 1B). However, after suctioning and removing the blood, no varices, erosions, ulcers, or vascular ectasia were observed in the stomach (Fig 1C) or esophagus (Fig 1D). The duodenum remained intact. The patient denied having had a nosebleed but continued to expectorate blood.

**Figure 2:**
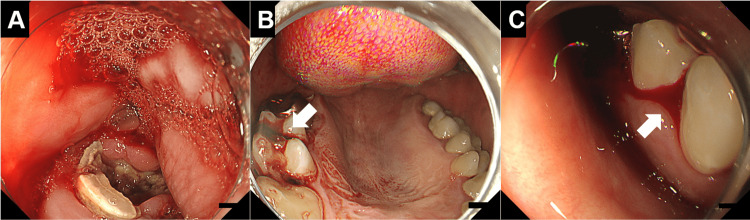
Esophagogastroduodenoscopy images of the oral cavity Dental caries were observed through endoscopy (A). Additionally, fresh blood continued to accumulate in the oral cavity. Aspiration of the blood facilitated a comprehensive examination of the gums, revealing persistent bleeding from the gingiva (B, C; arrows), thereby confirming a definitive diagnosis of gingival hemorrhage.

Re-examining the oral cavity through endoscopy revealed dental caries (Fig 2A). Furthermore, fresh blood continued accumulating in the oral cavity. Aspiration of fresh blood enabled a thorough examination of the gums, revealing persistent bleeding from the gingiva (Figures 2B-2C; arrows, Video 1), thereby confirming the definitive diagnosis of gingival bleeding. The blood observed in the esophagus and stomach was presumed to be swallowed blood originating from the oral cavity. The dentist examined the patient and noted swelling of the gums and gingival bleeding, leading to a diagnosis of periodontitis. The dental examination revealed no neoplastic lesions or vascular abnormalities, including aneurysms, in the oral cavity. The dentist applied gauze for compression hemostasis to manage the gingival bleeding. Since the procedure was conducted in the emergency department, only emergency hemostatic measures were taken. As the bleeding was caused by periodontitis, periodontal treatment at a dental clinic and extraction of the decayed tooth were recommended. The patient did not return to our dental department after that, and his subsequent clinical course remains unknown.

**Video 1:** Gingival bleeding observed during esophagogastroduodenoscopy.

## Discussion

When a patient with a history of heavy alcohol consumption presents with hematemesis, several differential diagnoses should be considered. These include rupture of esophageal or gastric varices and Mallory-Weiss syndrome [7-9]. Portal hypertension can result in vascular abnormalities in the stomach, including gastric and diffuse antral vascular ectasias. These conditions may contribute to anemia in patients with liver cirrhosis. Therefore, timely endoscopic intervention is critical for patients presenting with hematemesis.

In our patient, despite a comprehensive endoscopic examination of the esophagus, stomach, and duodenum, the source of bleeding remained elusive. In such cases, bleeding from sources outside the gastrointestinal tract, such as epistaxis, should be considered [10,11]. In a previous study, upper gastrointestinal hemorrhage was assessed via esophagogastroduodenoscopy in 461 patients with liver cirrhosis, which showed that epistaxis rather than upper gastrointestinal hemorrhage was the source of bleeding in 20 patients (4.3%) [11]. Although significant attention is typically given to bleeding from the esophagus, stomach, and duodenum, it is crucial to recognize that bleeding can also originate from non-gastrointestinal sites like the nasal and oral cavities.

Heavy alcohol consumption is associated with an increased risk of periodontal disease and dental caries [4-6]. Alcohol can cause dehydration and dry mouth by reducing saliva flow, which is a critical factor for neutralizing acids and protecting teeth from decay. Alcohol abuse often results in poor oral hygiene and a compromised immune system, promoting bacterial proliferation, subsequent gum inflammation, and tooth decay. Chronic alcohol consumption is also linked to nutritional deficiencies that further compromise oral health. Our patient had dental caries and periodontitis, with the latter being the cause of bleeding.

Recognizing that patients with liver cirrhosis face a higher overall bleeding risk compared to healthy individuals is essential [9]. Hepatic dysfunction can impair the synthesis of clotting factors, while thrombocytopenia, frequently observed in liver disease, decreases the platelet count essential for effective hemostasis. This interplay heightens the risk of bleeding complications, especially in individuals with a history of alcohol consumption. Clinicians must consider these factors when evaluating and managing hematemesis in patients with liver disease.

To our knowledge, no specific reports have directly investigated the frequency of hematemesis due to oral problems such as gingivitis. Given the unusual presentation of hematemesis from an oral source, this case underscores the importance of comprehensive evaluation beyond the gastrointestinal tract in patients with suspected upper gastrointestinal bleeding. In clinical practice, emphasis is often placed on diagnosing common causes, such as variceal bleeding, peptic ulcers, or Mallory-Weiss tears, especially in individuals with a history of heavy alcohol use. However, when these typical sources are excluded through careful history taking, symptom assessment, physical examination, and endoscopic examination, clinicians must broaden their differential diagnoses to include less common etiologies, such as bleeding from the oral or nasal cavity [12].

## Conclusions

This case underscores the importance of considering sources of bleeding outside the gastrointestinal tract, particularly oral bleeding, in patients with a history of alcohol consumption, given their increased risk of oral health problems. Endoscopic examination of the oral and pharyngolaryngeal regions is crucial because it allows the detection of various lesions in these areas. Furthermore, clinicians should maintain a high index of suspicion for oral causes of bleeding in patients presenting with hematemesis, integrating oral examinations consistently into their diagnostic workup. Early recognition of oral health issues may facilitate timely management and potentially prevent life-threatening bleeding events.
